# Detecting Carbapenemases in Animal and Food Samples by Droplet Digital PCR

**DOI:** 10.3390/antibiotics11121696

**Published:** 2022-11-25

**Authors:** Maria Carelli, Francesca Griggio, Marina Mingoia, Cristiana Garofalo, Vesna Milanović, Nicola Pozzato, Francesca Leoni, Laura Veschetti, Giovanni Malerba, Angela Sandri, Cristina Patuzzo, Serena Simoni, Maria M. Lleo, Carla Vignaroli

**Affiliations:** 1 Department of Diagnostics and Public Health, University of Verona, 37134 Verona, Italy; 2 Centro Piattaforme Tecnologiche, University of Verona, 37134 Verona, Italy; 3 Department of Biomedical Sciences and Public Health, Polytechnic University of Marche, 60121 Ancona, Italy; 4 Department of Agricultural, Food and Environmental Sciences, Polytechnic University of Marche, 60121 Ancona, Italy; 5 Laboratorio di Diagnostica Clinica e Sierologia di Piano, Istituto Zooprofilattico Sperimentale delle Venezie, 37060 Buttapietra, Italy; 6 Laboratorio Nazionale di Riferimento (LNR) per il Controllo delle Contaminazioni Batteriche dei Molluschi Bivalvi, Istituto Zooprofilattico Sperimentale dell’Umbria e delle Marche “Togo Rosati”, 60121 Ancona, Italy; 7 Department of Neurosciences, Biomedicine and Movement Sciences, University of Verona, 37134 Verona, Italy; 8 Department of Life and Environmental Sciences, Polytechnic University of Marche, 60121 Ancona, Italy

**Keywords:** carbapenemases, droplet digital PCR, multidrug resistance, animal samples, food samples

## Abstract

Background: The presence of carbapenemase-producing bacteria (CPB) in animal hosts and along the food chain may result in the development of reservoirs for human infections. Several CPB strains isolated from animals have been reported, suggesting that transmission and dissemination of the corresponding genes between humans and animals may occur. Animal and food samples have complex backgrounds that hinder the detection of CPB present in low concentrations by standard detection procedures. Methods: We evaluated the possibility of detecting *bla*_KPC_, *bla*_VIM_, and *bla*_OXA-48_-like carbapenemases in 286 animal and food samples (faeces from farm and companion animals, raw meat, bivalve molluscs) by culture-based and standard molecular methods and by ddPCR. Results: The proposed ddPCR managed to detect the target genes, also in samples resulting negative to standard methods. While the presence of *bla*_KPC_ and *bla*_VIM_ was detected in few samples (~3%), one third of the samples (*n* = 94/283) carried different variants of *bla*_OXA-48_-like genes. Conclusion: A specific and sensitive method such as ddPCR could be suitable to evaluate the current veterinarian and environmental situation and to assess the dynamic transmission and persistence of CPB between animals and humans and vice versa.

## 1. Introduction

Detection of antibiotic-resistant bacteria in the environment has recently received increasing interest due to the potential health risk posed by bacteria associated to animals, livestock, or humans at the interface of wildlife and the anthropic world [1,2,3]. Indeed, the emergence of multidrug resistant (MDR) microorganisms in animal hosts and along the food chain, or by other routes such as water or other environmental contamination as well as through direct animal contact, may result in the development of MDR microbial reservoirs for potential human infections [2,3]. Although carbapenems are not routinely used in veterinary medicine, infection or colonization by carbapenemase-producing bacteria in livestock and companion animals are increasingly being reported [4,5,6,7,8]. This observation might indicate effects of selection pressure resulting from increased acquisition of such isolates via the environment or animal food, rising transmission from humans to their cohabitants, as well as developing transmission in veterinary hospitals [9,10,11,12]. There have even been reports of wildlife having acquired carbapenemase-producing bacteria, for example by contact with sewage, manure, or waste disposal sites [13].

Resistance to beta-lactam antibiotics, mediated by beta-lactamases and carbapenemases, is of particular concern and epidemiological relevance because the genes encoding these enzymes are frequently located on mobile genetic elements such as transposons, integrons, and plasmids and can be transferred horizontally between bacterial cells [14]. The active transfer of carbapenemase-encoding plasmids among bacterial strains, species, and genera has been documented as part of hospital outbreaks [14,15]. However, the role of environmental carbapenemase-producing bacteria (CPB) in human diseases has been described but not investigated in detail, and only a few reports exist on antibiotic resistance of isolates from non-human origin [10,16,17,18,19]. Although the occurrence of CPB has been scarcely reported in animal hosts and their epidemiology remains quite unknown, their incidence seems to be increasing as reported by the recently established European Antimicrobial Resistance Surveillance network in veterinary medicine (EARS-Vet) [20].

MDR and extended-spectrum beta-lactamase strains (ESBL) have been found in samples of soft tissues, urinary tract infections, the respiratory tract, the genital tract, wounds, and faeces of dogs, cats, and horses admitted to veterinary clinics worldwide [10,21,22,23]. Interestingly, isolates from companion animals, horses, and humans obtained during a recent German screening [24] shared the same characteristics: presence of ESBL, CPB, and plasmid-encoded quinolone resistance genes. It has been speculated that this coincidence of common features might prove the active transmission and dissemination of MDR genes among animal and human populations, supporting the hypothesis that wildlife commonly harbours MDR microorganisms [2].

Monitoring of CPB in food and animal samples is very challenging because these bacteria can be in low concentrations in complex environmental backgrounds such as faeces or farm samples, and/or occur in stressed conditions not allowing their recovery in culture [25]. For these reasons, it is necessary to look for the genes of interest by using highly sensitive molecular methods to assess the current situation, thoroughly and reliably monitor it in the future [26], and track dissemination routes between different settings.

Droplet digital PCR (ddPCR) is a recently developed molecular technique with the potential to have a substantial impact on microbiology research owing to its reproducibility, sensitivity, and low susceptibility to inhibitors [27]. This method has proved to be powerful for quantifying absolute numbers of DNA sequences, even at very low concentrations, and is therefore suitable for studying the prevalence of CPB strains in challenging samples including animal faeces and food. ddPCR technology may potentially alleviate or greatly reduce limitations associated with other PCR methods, such as difficulty in multiplexing and susceptibility to inhibitors that are frequent within environmental and animal samples [28,29,30,31]. ddPCR also provides direct standards-free quantification, unlike qPCR methods where an amplification efficiency curve is required for the specific absolute quantification of the nucleic acid [32]. Indeed, in the ddPCR reaction the sample is partitioned into 20,000 droplets, and, for each droplet, the fluorescence is measured at the end of the PCR-end point reaction. Droplets containing target sequences are scored as positive, droplets without a target are scored as negatives, and absolute template quantity is determined by Poisson statistical data analysis. Therefore, as absolute quantification by ddPCR does not require a calibration curve, it is less prone to laboratory- and operator-related errors [29,30].

In this study, after searching CPB and carbapenemase genes with standard culture and PCR methods, we have evaluated the feasibility of using ddPCR to search for *bla*_KPC_, *bla*_VIM_ and *bla*_OXA-48_-like carbapenemase genes in complex background matrices, including veterinarian and food samples, obtained in the Veneto and Marche regions in Italy.

## 2. Results

### 2.1. Detection of Carbapenem Resistant Bacteria Using Conventional Methods

During the first part of the study, we used standard culture methods to detect carbapenemase-resistant bacteria, followed by MIC (minimum inhibitory concentration) testing to evaluate carbapenem resistance, MHT (modified Hodge test) to assess carbapenemases activity, and standard PCR to detect genes encoding enzymes acting on carbapenems (Table 1).

From a total of 626 samples tested by culture method, 151 samples contained bacterial colonies growing on ertapenem-enriched medium and/or showing resistance to ertapenem in the MIC essays. Among them, only two showed carbapenemases activity on the basis of the MHT and tested positive for the presence of carbapenemases genes by standard PCR (Table 1). These two isolates were further identified by WGS.

In more detail, fifty-three samples of pig and cattle faeces were analysed by culture method and MIC: thirteen strains showed a MIC > 2 µg/mL (indicating resistance to ertapenem) but they resulted negative from the MHT.

Around 26% of the companion animals’ faeces (*n* = 77/295) harboured strains that were able to grow in presence of ertapenem. Among these, none showed carbapenemases activity and in no cases were carbapenemase genes detected by PCR.

Around 20% of meat samples (*n* = 12/58) were susceptible to ertapenem (MIC < 0.25 µg/mL). The 12 strains that were found to be resistant were further investigated with MHT and standard PCR, and only in one of them was the presence of *bla*_OXA-48_-like gene detected.

Forty-nine out of 220 mollusc samples growing at a low ertapenem concentration were investigated for MIC response values to this antibiotic and were screened using the MHT test. Only one of the isolates showed carbapenemases activity; the strain was identified by WGS and OrthoANI analysis as *Klebsiella michiganensis* (https://www.ezbiocloud.net/tools/ani) (accessed on 6 November 2022). The strain was resistant to ertapenem (MIC = 256 µg/mL) and test positive for the presence of the KPC-3 gene (BioProject ID PRJNA850894 accession number: JANFNZ000000000).

### 2.2. Optimization of Extraction and ddPCR Conditions

Different protocols were tested to extract genomic DNA with high purity. Nucleic acid extraction from bivalve molluscs was particularly laborious and only DNA from 27/220 samples reached the required quality for ddPCR application. A combination of DNeasy^®^ PowerSoil^®^ Pro (Qiagen, Hilden, Germany) and Tissue Lyser II (Qiagen, Hilden, Germany) provided the best results in terms of concentration and purity of DNA from bivalve molluscs.

After optimization of the protocol, a specific baseline was evaluated for each amplification round using positive and negative controls to discriminate background nonspecific amplification from positive droplets, to minimize false positive and false negative results.

Stringent quality controls were performed, including no amplification in NTC and negative control wells, exclusion of wells with less than 10,000 accepted droplets, evaluation of the number of accepted droplets per reaction distribution, and fluorescence amplitude of positive and negative droplets. The optimal annealing/extension temperature was set respectively at 55/58/60 °C for each of the investigated genes (*bla*_KPC_, *bla*_VIM_, *bla*_OXA-48_-like), and 45 s resulted in the best impact on the space between positive and negative droplets (Table 2).

To verify the diagnostic performance of ddPCR, we used 20 samples of human faeces previously screened for CPB able to grow in presence of ertapenem: ddPCR identified at least one of the *bla*_KPC_, *bla*_VIM_, and *bla*_OXA-48_-like genes in 85% (*n* = 17) of them.

Among these 17 positive samples, 58% (*n* = 10) carried *bla*_KPC_, 24% (*n* = 4) carried *bla*_VIM_, and all of them (*n* = 17) carried *bla*_OXA-48_-like genes. Meanwhile, 47% of them (*n* = 8) carried two of these carbapenemase genes, and 18% (*n* = 3) carried all three. As shown, a high number of positive droplets corresponds to positivity in conventional tests (Table 3).

After WGS, seven out of 20 isolates (35%) were found positive for the presence of carbapenemase genes: 36% of them (*n* = 5) carried *bla*_KPC_ genes, only 1 carried *bla*_VIM_, and only 1 carried the *bla*_OXA-48_-like gene. Other enzymes including TEM, CTX-M, SHV, CMY, and ADC-25 were also found among the isolates (Table 3).

### 2.3. Carbapenemase-Encoding Genes as Detected by ddPCR

The 283 samples from different sources (companion animal faeces, livestock meat and faeces, and molluscs) that presented with a sufficient amount of good-quality DNA were tested using ddPCR for the presence of *bla*_KPC_, *bla*_VIM_, and *bla*_OXA-48_-like carbapenemase-encoding genes.

The majority of samples did not harbour *bla*_KPC_ or *bla*_VIM_ resistance genes, whereas most of the livestock animal faeces and bivalve mollusc samples carried *bla*_OXA-48_-like resistance genes. In particular, *bla*_KPC_ genes were detected in seven samples (5%) of companion animals’ faeces, one (2%) of livestock animal faeces, and one (4%) of bivalve molluscs. No *bla*_KPC_ was detected in meat samples. Similarly, we found that five samples (3%) of companion animals’ faeces, three (6%) of livestock animal faeces, and one (2%) of meat samples showed positivity for *bla*_VIM_ genes. *bla*_OXA-48_-like genes were found in 27 (72.5%), 37 (18%), 14 (26%), and 16 (61.5%) samples of companion animals’ faeces, livestock animal faeces, meat, and bivalve molluscs, respectively (Table 4).

Statistical analysis showed that livestock faeces and bivalve mollusc samples were significantly more likely to carry at least one resistance gene when compared to all other sample types (*p* < 0.001). Moreover, it was statistically more likely to find a *bla*_OXA-48_-like resistance gene than a *bla*_KPC_ or *bla*_VIM_ gene (*p* < 0.001). *p* values resulting from the statistical analysis are reported in Tables S1 and S2.

## 3. Discussion

The increasing use of antibiotics in animals over the past century has led to the widespread transmission of CPB and carbapenemase-encoding genes between animals and humans [3]. The resistance to beta-lactam antibiotics, mediated by beta-lactamases and carbapenemases, is of particular concern and epidemiological relevance because the genes encoding these enzymes are frequently located on mobile genetic elements and can be transferred horizontally between bacterial cells [14].

Approximately 75% of antibiotics are not absorbed by the animals and are excreted from the body through faeces and urine, which can directly contaminate the surrounding environment. For this reason, animal farms are important reservoirs of CPB and carbapenemase-encoding genes constituting an emerging global threat for human and animal health [3]. In this scenario, monitoring the prevalence of CPB and carbapenemase-encoding genes becomes particularly important for estimating the level of risk they represent for human health and eventually controlling their diffusion among infected animals.

This is currently achieved using conventional culture-based and molecular methods. However, it is well known that in animal faeces and meat the presence of a high background of bacteria and eukaryotic DNA and/or PCR inhibitors co-extracted with DNA negatively affect PCR efficiency and quantification accuracy [26]. Moreover, bacteria, especially those present in low concentrations and not in optimal environmental conditions, might be in stress-response states and unrecoverable in culture [25]. As such, very sensitive molecular methods such as droplet digital PCR can represent valid alternatives to other molecular and standard culture methods for these types of samples [28,30].

In this study, the application of ddPCR to veterinarian samples included first an optimization process, both in terms of DNA quality (different protocols were tested to extract genomic DNA with high purity) and of protocols for adjustment in order to obtain results with coherent clusters of positive droplets well-separated from the negative background.

Within that process, optimization of melting temperature was attained by testing a temperature gradient from 52 °C to 62 °C to achieve best droplet separation. The optimal annealing/extension temperature was set respectively at 55/58/60 °C for each of the investigated genes (*bla*_KPC_, *bla*_VIM_, *bla*_OXA-48_-like). Moreover, extension times of 30–45–60 s were tested according to the thermal cycling protocol recommended by the manufacturer, and 45 s resulted in the best impact on the space between positive and negative droplets.

The optimal amount of DNA sample to load onto the cartridge was evaluated at 50 ng, to provide an optimal negative baseline and accurate detection of the target genes and to avoid primer dimers. We decided to reload the samples that produced fewer than three positive droplets, to increase the amount of tested DNA and the sensitivity of the ddPCR. As the amount of tested DNA increased, the sensitivity of the ddPCR increased. However, the sensitivity is also determined by other factors, especially by the sample homogeneity and the efficiency and purity of DNA extraction, which are crucial in complex samples such as faeces and non-mammalian tissue.

Comparing the results obtained using the ddPCR protocol with those acquired by standard methods, we observed that the numbers of positive samples for the three carbapenemase-encoding genes, and especially those of the OXA-48 family, were much higher than those obtained by the conventional methods. Although the prevalence of the KPC and VIM genes was low (2–4% for KPC and 3–6% for VIM), we found a high number of samples positive for the presence of *bla*_OXA-48_-like genes, respectively 72.5%, 26%, and 61.5% for faeces of livestock animals, meat, and molluscs collected in the Marche region, and 18% prevalence in faeces of companion animals collected in the Veneto region. Livestock faeces and bivalve mollusc samples were significantly more likely to carry at least one resistance gene when compared with all other sample types (*p* < 0.001). Certain studies in Europe have indicated a low prevalence of CPB strains in livestock [10,33,34]. However, they were performed in Switzerland and the Netherlands, where the prevalence of CPB in humans is also reported to be lower than in Southern Europe, including Italy. Nonetheless, studies on CPB in animals and seafood have rarely been performed and prevalence data are still lacking.

In our study, it was statistically more likely to find a *bla*_OXA-48_-like resistance gene than *bla*_KPC_ or *bla*_VIM_ genes (*p* < 0.001), in accordance with previous reports of high *bla*_OXA-48_-like prevalence in animals (dogs, cats, and horses) and humans [10,11,12]. It is noteworthy that some samples in our study presented more than one type of carbapenemase: seven out of the 18 KPC- and VIM- positive samples also presented the OXA-48 like enzyme, while in two samples KPC and VIM-encoding genes were detected.

The ddPCR technique also showed some limitations, including the requirement of protocol optimization both in the experimental setup and the process, e.g., for annealing temperature and threshold. Moreover, this protocol is not intended for multiplexing, as the annealing temperature that led to the best droplet separation with less rain was different for the investigated genes. In addition, the primers used in this study amplify gene families, so the sequence variability of the genome region lying between each primer pair makes it difficult to design a specific probe. Nevertheless, this protocol could lead to the detection of a huge number of carbapenemase-encoding genes, including those not yet described in databases. To evaluate the performance of the ddPCR protocol in detection of CPB in complex samples, we analysed 20 samples of human faeces previously screened for CPB by culturing methods. Although the culturing method cannot be compared with a quantitative method, it should be highlighted that when the sample was positive for the presence of CPB, ddPCR showed a very high number of positive droplets (>1000).

After adequate optimization of our ddPCR protocol, the results obtained in this study confirm the adequacy of this method as an approach for CPB detection in veterinarian and food samples, which are known to be complex both in terms of DNA diversity and contaminants as well as having low concentrations of target genes. To prevent the occurrence of CPB and carbapenemase-encoding genes and their spread in wildlife, food-producing and companion animals should be a major public health priority to protect individuals with direct exposure and consumers [3]. Emergence and spread of CPB in livestock and companion animals may represent an underestimated threat to public health, and fast and sensitive methods are required to systematically screen food and veterinary samples. Droplet digital PCR appears suitable for CPB detection in complex samples, providing faster, more sensitive, and better reproducible data than conventional methods.

## 4. Materials and Methods

### 4.1. Areas Considered in the Study

Samples were obtained from different animal farms located in the Veneto and Marche regions in Eastern Italy. Mollusc samples were taken from the Adriatic Sea coast in the Ancona province (Marche region).

### 4.2. Sampling

#### 4.2.1. Animal Samples

- Faecal samples from farm animals. Fifty-tree samples of animal faeces were collected from 7 small- and medium-scale pig and cattle farms located in the Marche region. For each sample about 60 g of faeces were obtained from a mix of 3–5 stool samples collected from apparently healthy finishing pigs and beef cattle. After nucleic acid extraction, 51 samples reached sufficient quantity and purity for downstream application (see Section 4.4).
- Faecal samples from companion animals. A total of 295 faecal or litter samples from mammals (dogs, cats, other mammals) and ornamental birds (finches, psittacides, other birds) received for diagnostic purposes at the Verona laboratory of Istituto Zooprofilattico delle Venezie (IZSVe) were selected. Upon arrival, samples were plated in culture medium containing ertapenem, and 2 g of each sample were recovered and stored at −20 °C until further processing. After nucleic acid extraction, 152 samples reached sufficient quantity and purity for downstream application (see Section 4.4).

#### 4.2.2. Food Samples

- Raw meat. Fifty-eight samples of raw pork and beef meat were collected at the slaughterhouses of the seven above-mentioned farms. Under sterile conditions, 100 g of meat were sampled, immediately stored at 4 °C and processed within 24 h. After nucleic acid extraction, fifty-four samples reached sufficient quantity and purity for downstream application (see Section 4.4).
- Bivalve molluscs. Two hundred and twenty samples were collected from natural beds along the Adriatic Sea coast in the Ancona province. Harvesting areas more impacted by faecal contamination were selected for this study, based on historical microbiological quality data for the area, and sources and types of faecal contamination in close proximity. Sampling was performed in two different seasons (winter and summer) according to the variation in filter-feeding activity of bivalve molluscs. After nucleic acid extraction, 27 samples reached levels of quantity and purity required for downstream application (see Section 4.4).

#### 4.2.3. Human Samples

Twenty human faecal samples collected for diagnostic purposes and testing positive for CPB by culture and standard PCR methods were assessed to evaluate the suitability of ddPCR for detecting carbapenemase-encoding genes using DNA directly extracted from complex samples. The strains isolated from the samples were also sequenced to confirm the results obtained. Faecal or rectal swab samples were obtained from patients attending the outpatient clinic of the Infectious Disease Department of the Polytechnic University of Marche and from outpatients and inpatients of Ancona Hospitals.

### 4.3. Detection of CPB by Conventional Methods

Aliquots of each faecal and food sample (~10 g) were homogenized in 90 mL of sterilized peptone water (10.0 g/L) for 2 min at 260 rpm using the Stomacher 400 circulator machine (International PBI, Milan, Italy). Then, 100 µL from each homogenate were inoculated onto 10 mL of Luria–Bertani (LB) broth supplemented with 0.12 µg/mL ertapenem (Merck KGaA, Darmstadt, Germany). After incubation at 37 °C for 24 h, the tubes showing turbidity were ten-fold serially diluted and 100 µL of the appropriate dilutions were spread on MacConkey Agar containing ertapenem (0.12 µg/mL) and incubated at 37 °C for 24 h.

The presumptive carbapenem-resistant colonies were selected and analysed for their carbapenemase activity using MHT [35] and/or MIC determination against carbapenems following EUCAST guidelines.

CPB were evaluated for the class of carbapenemases they produced (KPC, VIM, and OXA-48-like types), through standard PCR using specific primers [36].

### 4.4. DNA Extraction and Purification

A 1.5 mL aliquot of each sample was processed for direct extraction of bacterial DNA with a commercial kit suitable to the type of sample under examination. In particular, bacterial DNA was extracted from raw meat using a PowerFood microbial DNA isolation kit—MoBio Laboratories; from human stool samples using a QIAamp fast DNA stool ini kit (Qiagen, Hilden, Germany); from animal stool samples using an E.Z.N.A. soil DNA kit—VWR and DNeasy^®^ PowerSoil^®^ Pro kit (Qiagen, Hilden, Germany), following the manufacturers’ instructions. For the bivalve molluscs (clams), bacterial DNA was extracted from the digestive gland or from clam homogenates. Nuclisens^®^ lysis buffer (Biomerieux, Lyon, France) and a Nuclisens^®^ magnetic extraction kit (Biomerieux, Lyon, France) were used respectively for extraction and purification of DNA from the digestive gland, according to the manufacturer’s instructions. From the whole clam homogenates, DNA was extracted using the Tissue Lyser II (Qiagen, Hilden, Germany) and the DNeasy^®^ PowerSoil^®^ Pro kit (Qiagen, Hilden, Germany) according to the manufacturer’s instructions. DNA purity and yield were assessed spectrophotometrically (Qubit, Thermo Fisher Scientific, Waltham, MA, USA) and fluorometrically (Nanodrop, Thermo Fisher Scientific, Waltham, MA, USA).

### 4.5. Primers for Droplet Digital PCR

Droplet digital PCR was employed to determine directly the presence of *bla*_KPC_, *bla*_VIM_, and *bla*_OXA-48_-like genes in samples. The primer sequences and product sizes [37] are presented in Table 5.

Primers were first tested in silico using the OligoAnalyzer tool available online on the IDT platform (https://eu.idtdna.com, accessed on 22 June 2020) to evaluate the possible formation of hairpins, homodimers, and heterodimers between the forward and reverse primers. Moreover, the primer sequences were analysed with BLAST against the nr database (accession date: 4 December 2020) with default parameters to verify the presence of the searched genes and non-specific amplicons, and were screened in silico against all the OXA sequences (*n* = 439, accession date: 2 December 2020) in the comprehensive antibiotic resistance database (CARD). After this analysis, we observed that the *bla*_OXA-48_ primer pair is able to amplify a great number of *bla*_OXA_-variant encoding genes (OXA-484, OXA-48, OXA-439, OXA-438, OXA-416, OXA-405, OXA370, OXA-252, OXA-247, OXA-245, OXA-244, OXA-232, OXA-204, OXA-199, OXA-181, OXA-163, OXA-162, OXA-54, OXA-535, OXA-436, OXA-293).

### 4.6. Droplet Digital PCR

ddPCR reactions were conducted in a QX200 droplet digital PCR system (Bio-Rad, Hercules, CA, USA) with QX200 ddPCR EvaGreen Supermix (Bio-Rad, Hercules, CA, USA). To increase cluster separation, the annealing/extension time and temperature were both optimized. Melting temperature optimization was achieved by testing a temperature gradient from 52 °C to 62 °C to achieve the best droplet separation, and extension times of 30–45–60 s were tested according to the thermal cycling protocol recommended by the manufacturer.

The ddPCR reaction mixture was composed of 11 μL ddPCR EvaGreen Supermix 2X (Bio-Rad, Hercules, CA, USA), 100 nM of each primer, 50 ng template, and Ultrapure DNase/RNase free distilled water (Thermo Fisher Scientific, Waltham, MA, USA) to reach a final volume of 22 μL. Following this step, 20 μL of each reaction mix and 70 μL of droplet generator oil were transferred into a droplet generator cartridge (Bio-Rad, Hercules, CA, USA). To perform the droplet generation, the cartridge was placed into a QX200 droplet generator (Bio-Rad, Hercules, CA, USA).

The droplet-partitioned samples were transferred into a 96-well plate that was heat-sealed with a PCR plate heat-seal foil using the Px1 PCR plate sealer (Bio-Rad, Hercules, CA, USA). The plate was placed in a SimpliAmp thermal cycler (Applied Biosystems, Waltham, MA, USA) for PCR amplification, following the cycling protocol reported in Table 2. The amplified samples were transferred to a droplet reader (Bio-Rad, Hercules, CA, USA) for data acquisition.

In each experiment, a positive control and two negative controls were included. As positive controls, we used 50 ng of DNA extracted from a non-CPB isolate mixed with a small amount of DNA (0.0001 ng) extracted from CPB reference isolates (previously confirmed as CPB by culture methods and RT-PCR). This was carried out to simulate a sample containing a small amount of carbapenemase-encoding genes and a large amount of background DNA, and to load into each cartridge well the same amount of DNA to ensure a similar baseline of negative droplets. The negative controls were a no-template sample (NTC) to test the absence of contaminants, and 50 ng DNA extracted from a non-CPB isolate to evaluate false positive droplets. Data were analysed using the QuantaSoft™ Software version 1.7 (Bio-Rad, Hercules, CA, USA).

### 4.7. Whole Genome Sequencing (WGS) Analysis

Presence of *bla*-genes in control strains from human samples was evaluated by WGS analysis. WGS was performed by MicrobesNG Service (https://microbesng.com/, accessed on 20 October 2021) using the Illumina Miseq short-read technology (2 × 250 paired ends) in combination with Oxford Nanopore long reads. The assembled and annotated genome provided by MicrobesNG Service was further analysed using ResFinder v.3.2, available at the Center for Genomic Epidemiology (https://www.genomicepidemiology.org/, accessed on 15 November 2021).

### 4.8. Statistical Analysis

Statistical analysis was carried out by ddPCR to compare the results obtained for the presence of antibiotic-resistance genes in the different samples, using Fisher’s exact test followed by Bonferroni correction for multiple comparisons. Samples that were not tested for all antibiotic resistance genes or had undetermined results were excluded from this analysis. R version 4.0.4 was used for statistical analysis and for visualization of results.

## 5. Conclusions

Monitoring the prevalence of carbapenemase-producing bacteria in farm animals and the food chain is important for estimating the level of risk they represent for human health, and eventually controlling their diffusion. This is currently achieved using conventional culture-based and molecular methods with low efficiency and accuracy. As such, very sensitive molecular methods such as droplet digital PCR can represent valid alternatives to other molecular and standard culture methods for these types of samples.

## Figures and Tables

**Table 1:** Samples tested for carbapenem resistance by conventional methods (culture in ertapenem-enriched medium, MIC test, MHT, PCR). The number of analysed and positive samples for each test by sample type is reported in the table.

	Total Samples Tested (N = 626)	Culture Method	MHT	Standard PCR
Faeces (livestock animals)	53	13	-	-
Meat (livestock animals)	58	12	1	1
Faeces (companion animals)	295	77	-	-
Bivalve molluscs	220	49	1	1

**Table 2:** ddPCR cycling protocol.

Temperature	Time	Cycles (N°)
95 °C	5 min	1
95 °C	30 s	40
55 °C–58 °C–60 °C (respectively for *bla*_KPC_*, bla*_VIM_*, bla*_OXA-48_-like detection)	45 s	
4 °C	5 min	1
90 °C	5 min	1
4 °C	hold	

**Table 3:** Carbapenemase-positive samples of human feces as determined by culture method (MacConkey Agar containing ertapenem), by ddPCR and by WGS analysis.

Sample ID	ddPCR						Carbapenemase Producing Isolate	Bacterial Species	*bla* Genes
	*bla*_KPC_ Result Positive Droplets		*bla*_VIM_ Result Positive Droplets		*bla*_OXA-48_-like Result Positive Droplets				
A	+	16,500	+	5	+	36	+	*K. pneumoniae*	*KPC*, *TEM*, *CTX-M*, *SHV*
B	+	2356	−	3	+	19	+	*K. pneumoniae*	*KPC*, *TEM*, *CTX-M*, *SHV*
C	+	16,662	−	3	+	11	+	*K. pneumoniae*	*KPC*, *TEM*, *CTX-M*, *SHV*
D	−	3	+	13,514	+	37	+	*P. oleovorans*	*VIM*
E	+	857	−	0	+	8	+	*K. pneumoniae*	*KPC*, *TEM*, *CTX-M*, *SHV*
F	+	15,646	+	10,471	+	38	+	*K. pneumoniae*	*KPC*, *TEM*, *CTX-M*, *SHV*
G	+	5	−	0	+	31	−	*K. pneumoniae*	*TEM*, *CTX-M*, *SHV*
H	+	4	−	1	+	13	+	*A. baumannii*	*OXA-66*, *OXA-72*, *ADC-25*
I	−	2	−	0	+	10	−	*K. pneumoniae*	*CTX-M*, *SHV*
J	−	1	−	1	−	2	−	*H. alvei*	*SHV*
K	−	0	−	0	−	2	−	*K. pneumoniae*	*TEM*, *CTX-M*, *SHV*
L	+	80	+	14	+	56	−	*K. pneumoniae*	*SHV*
M	+	17	−	0	+	13	−	*S. liquefaciens*	-
N	−	2	−	0	+	8	−	*K. pneumoniae*	*TEM*, *CTX-M*, *SHV*
O	+	8	−	0	+	20	−	*C. freundii*	-
P	−	0	−	1	−	2	−	*E. cloacae*	-
Q	−	0	−	0	+	7	−	*E. cloacae*	-
R	−	0	−	0	+	18	−	*C. freundii*	*CMY*
S	−	3	−	1	+	20	−	*S. maltophila*	-
T	−	3	−	0	+	28	−	*M. morganii*	-

**Table 4:** Antibiotic resistance genes detected by ddPCR by sample type and by tested resistance genes.

		*blaKPC*		*blaVIM*		*blaOXA-48*-like	
	N. of Samples with at Least 1 Resistance Gene	Samples Tested (N = 281)	Detected	Samples Tested (N = 280)	Detected	Samples Tested (N = 283)	Detected
Faeces (livestock animals)	38	51	1	51	3	51	37
Meat (livestock animals)	14	54	-	54	1	54	14
Faeces (companion animals)	34	149	7	149	5	152	27
Bivalve molluscs	17	27	1	26	-	26	16

**Table 5:** Primers used for detecting carbapenemase-encoding genes by ddPCR.

Gene	Primer Sequence	Product Size	Reference
*bla*_OXA-48_-like	OXA48_F: 5′ TGTTTTTGGTGGCATCGAT 3′	177 bp	[25]
	OXA48_R: 5′ GTAAMRATGCTTGGTTCGC 3′		
*bla*_KPC_	KPC_F: 5′ CAGCTCATTCAAGGGCTTTC 3′	155 bp	
	KPC_R: 5′ GGCGGCGTTATCACTGTATT 3′		
*bla*_VIM_	VIM_F: 5′ TCCGTGATGGTGATGAGT 3′	262 bp	
	VIM_R: 5′ GCTCGATGAGAGTCCTTCTA 3′		

## Data Availability

The WGS data presented in this study are openly available in BioProject ID PRJNA850894 accession number: JANFNZ000000000.

## Supplementary Materials

The following supporting information can be downloaded at: https://www.mdpi.com/article/10.3390/antibiotics11121696/s1, Table S1: results of the statistical analysis by sample type; Table S2: results of the statistical analysis by resistance genes.

## References

[1] Aslam B., Khurshid M., Arshad M.I., Muzammil S., Rasool M., Yasmeen N., Shah T., Chaudhry T.H., Rasool M.H., Shahid A. (2021). Antibiotic Resistance: One Health One World Outlook. Front. Cell Infect. Microbiol..

[2] Swift B.M., Bennett M., Waller K., Dodd C., Murray A., Gomes R.L., Humphreys B., Hobman J.L., Jones M.A., Whitlock S.E. (2019). Anthropogenic environmental drivers of antimicrobial resistance in wildlife. Sci. Total Environ..

[3] Xu C., Kong L., Gao H., Cheng X., Wang X. (2022). A Review of Current Bacterial Resistance to Antibiotics in Food Animals. Front. Microbiol..

[4] Adams R.J., Kim S.S., Mollenkopf D.F., Mathys D.A., Schuenemann G.M., Daniels J.B., Wittum T.E. (2018). Antimicrobial-resistant Enterobacteriaceae recovered from companion animal and livestock environments. Zoonoses Public Health.

[5] Cheng V.C.C., Wong S.C., Wong S.C.Y., Ho P.L., Yuen K.Y. (2018). Control of Carbapenemase-producing Enterobacteriaceae: Beyond the Hospital. EClinicalMedicine.

[6] Poirel L., Bercot B., Millemann Y., Bonnin R.A., Pannaux G., Nordmann P. (2012). Carbapenemase-producing Acinetobacter spp. in Cattle, France. Emerg. Infect. Dis..

[7] Fischer J., Rodríguez I., Schmoger S., Friese A., Roesler U., Helmuth R., Guerra B. (2013). Salmonella enterica subsp. enterica producing VIM-1 carbapenemase isolated from livestock farms. J. Antimicrob. Chemother..

[8] Fischer J., José M.S., Roschanski N., Schmoger S., Baumann B., Irrgang A., Friese A., Roesler U., Helmuth R., Guerra B. (2017). Spread and persistence of VIM-1 Carbapenemase-producing Enterobacteriaceae in three German swine farms in 2011 and 2012. Vet. Microbiol..

[9] Ljungquist O., Ljungquist D., Myrenas M., Ryden C., Finn M., Bengtsson B. (2016). Evidence of household transfer of ESBL-/pAmpC-producing Enterobacteriaceae between humans and dogs—A pilot study. Infect. Ecol. Epidemiol..

[10] Köck R., Daniels-Haardt I., Becker K., Mellmann A., Friedrich A.W., Mevius D., Schwarz S., Jurke A. (2018). Carbapenem-resistant Enterobacteriaceae in wildlife, food-producing, and companion animals: A systematic review. Clin. Microbiol. Infect..

[11] Silva J.M.D., Menezes J., Marques C., Pomba C.F. (2022). Companion Animals-An Overlooked and Misdiagnosed Reservoir of Carbapenem Resistance. Antibiotics.

[12] Bonardi S., Pitino R. (2019). Carbapenemase-producing bacteria in food-producing animals, wildlife and environment: A challenge for human health. Ital. J. Food Saf..

[13] Mairi A., Pantel A., Sotto A., Lavigne J.P., Touati A. (2018). OXA-48-like carbapenemases producing Enterobacteriaceae in different niches. Eur. J. Clin. Microbiol. Infect. Dis..

[14] León-Sampedro R., DelaFuente J., Díaz-Agero C., Crellen T., Musicha P., Rodríguez-Beltrán J., de la Vega C., Hernández-García M., López-Fresneña N., Ruiz-Garbajosa P. (2021). Pervasive transmission of a carbapenem resistance plasmid in the gut microbiota of hospitalized patients. Nat. Microbiol..

[15] Gong L., Tang N., Chen D., Sun K., Lan R., Zhang W., Zhou H., Yuan M., Chen X., Zhao X. (2020). A Nosocomial Respiratory Infection Outbreak of Carbapenem-Resistant Escherichia coli ST131 With Multiple Transmissible bla KPC-2 Carrying Plasmids. Front. Microbiol..

[16] Abraham S., Wong H.S., Turnidge J., Johnson J.R., Trott D.J. (2014). Carbapenemase-producing bacteria in companion animals: A public health concern on the horizon. J. Antimicrob. Chemother..

[17] Guerra B., Fischer J., Helmuth R. (2014). An emerging public health problem: Acquired carbapenemase-producing microorganisms are present in food-producing animals, their environment, companion animals and wild birds. Vet. Microbiol..

[18] Sacher-Pirklbauer A., Klein-Jobstl D., Sofka D., Blanc-Potard A.B., Hilbert F. (2021). Phylogenetic Groups and Antimicrobial Resistance Genes in Escherichia coli from Different Meat Species. Antibiotics.

[19] Treskova M., Kuhlmann A., Freise F., Kreienbrock L., Brogden S. (2022). Occurrence of Antimicrobial Resistance in the Environment in Germany, Austria, and Switzerland: A Narrative Review of Existing Evidence. Microorganisms.

[20] Mader R., Damborg P., Amat J.-P., Bengtsson B., Bourély C., Broens E.M., Busani L., Crespo-Robledo P., Filippitzi M.-E., Fitzgerald W. (2021). Building the European Antimicrobial Resistance Surveillance network in veterinary medicine (EARS-Vet). Eurosurveillance.

[21] Belas A., Menezes J., Gama L.T., Pomba C. (2020). Sharing of Clinically Important Antimicrobial Resistance Genes by Companion Animals and Their Human Household Members. Microb. Drug Resist..

[22] Khalifa H.O., Oreiby A.F., El-Hafeez A.A.A., Okanda T., Haque A., Anwar K.S., Tanaka M., Miyako K., Tsuji S., Kato Y. (2020). First Report of Multidrug-Resistant Carbapenemase-Producing Bacteria Coharboring mcr-9 Associated with Respiratory Disease Complex in Pets: Potential of Animal-Human Transmission. Antimicrob. Agents Chemother..

[23] Van den Bunt G., Fluit A.C., Spaninks M.P., Timmerman A.J., Geurts Y., Kant A., Scharringa J., Mevius D., Wagenaar J.A., Bonten M.J.M. (2020). Faecal carriage, risk factors, acquisition and persistence of ESBL-producing Enterobacteriaceae in dogs and cats and co-carriage with humans belonging to the same household. J. Antimicrob. Chemother..

[24] Schmiedel J., Falgenhauer L., Domann E., Bauerfeind R., Prenger-Berninghoff E., Imirzalioglu C., Chakraborty T. (2014). Multiresistant extended-spectrum beta-lactamase-producing Enterobacteriaceae from humans, companion animals and horses in central Hesse, Germany. BMC Microbiol..

[25] Bodor A., Bounedjoum N., Vincze G.E., Kis E., Laczi K., Bende G., Szilágyi Á., Kovács T., Perei K., Rákhely G. (2020). Challenges of unculturable bacteria: Environmental perspectives. Rev. Environ. Sci. Biotechnol..

[26] Hedman J., Radstrom P. (2013). Overcoming inhibition in real-time diagnostic PCR. Methods Mol. Biol..

[27] Cremonesi P., Garofalo C., Picozzi C., Castiglioni B., Mangieri N., Milanović V., Osimani A., Aquilanti L. (2022). Development of quantitative real-time PCR and digital droplet-PCR assays for rapid and early detection of the spoilage yeasts Saccharomycopsis fibuligera and Wickerhamomyces anomalus in bread. Food Microbiol..

[28] Anderson R.E.V., Boerlin P. (2020). Carbapenemase-producing Enterobacteriaceae in animals and methodologies for their detection. Can. J. Vet. Res..

[29] Cave L., Brothier E., Abrouk D., Bouda P.S., Hien E., Nazaret S. (2016). Efficiency and sensitivity of the digital droplet PCR for the quantification of antibiotic resistance genes in soils and organic residues. Appl. Microbiol. Biotechnol..

[30] Cristiano D., Peruzy M., Aponte M., Mancusi A., Proroga Y., Capuano F., Murru N. (2021). Comparison of droplet digital PCR vs real-time PCR for Yersinia enterocolitica detection in vegetables. Int. J. Food Microbiol..

[31] Pendergraph D.P., Ranieri J., Ermatinger L., Baumann A., Metcalf A.L., DeLuca T.H., Church M.J. (2021). Differentiating Sources of Fecal Contamination to Wilderness Waters Using Droplet Digital PCR and Fecal Indicator Bacteria Methods. Wilderness Environ. Med..

[32] Lee C., Kim J., Shin S.G., Hwang S. (2006). Absolute and relative QPCR quantification of plasmid copy number in Escherichia coli. J. Biotechnol..

[33] Ceccarelli D., van Essen-Zandbergen A., Veldman K.T., Tafro N., Haenen O., Mevius D.J. (2017). Chromosome-Based blaOXA-48-Like Variants in Shewanella Species Isolates from Food-Producing Animals, Fish, and the Aquatic Environment. Antimicrob. Agents Chemother..

[34] Stephan R., Sarno E., Hofer E., Nuesch-Inderbinen M.T., Hachler H. (2013). Lack of evidence so far for carbapenemase-producing Enterobacteriaceae in food-producing animals in Switzerland. Schweiz. Arch. Tierheilkd..

[35] Zahedi Bialvaei A., Dolatyar Dehkharghani A., Asgari F., Shamloo F., Eslami P., Rahbar M. (2021). Modified CIM test as a useful tool to detect carbapenemase activity among extensively drug-resistant Klebsiella pneumoniae, Escherichia coli and Acinetobacter baumannii. Ann. Microbiol..

[36] Poirel L., Walsh T.R., Cuvillier V., Nordmann P. (2011). Multiplex PCR for detection of acquired carbapenemase genes. Diagn. Microbiol. Infect. Dis..

[37] Makowska N., Philips A., Dabert M., Nowis K., Trzebny A., Koczura R., Mokracka J. (2020). Metagenomic analysis of beta-lactamase and carbapenemase genes in the wastewater resistome. Water Res..

