# Supplementary material for: Detecting Carbapenemases in Animal and Food Samples by Droplet Digital PCR
Source: Antibiotics (Basel). 2022 Nov 25;11(12):1696. doi: 10.3390/antibiotics11121696 (PMC9774140; doi:10.3390/antibiotics11121696)
Supplement: Supplementary file 1 [file antibiotics-11-01696-s001.zip › antibiotics-2050638-supplementary.pdf]

Table S1. Results of the statistical analysis by sample type

| Sample type | p-value |
|---|---|
| All | <2,20E-22 |
| Faeces (livestock animals) vs Faeces (companion animals) | <2,20E-16 |
| Faeces (livestock animals) vs Meat (livestock animals) | 1,44E-11 |
| Faeces (livestock animals) vs Bivalve molluscs | 0,0086 |
| Faeces (companion animals) vs Meat (livestock animals) | 0,4 |
| Faeces (companion animals) vs Bivalve molluscs | 4,45E-05 |
| Meat (livestock animals) vs Bivalve molluscs | 0,002 |

Table S2. Results of the statistical analysis by resistance genes

| Resistance genes | p-value |
|---|---|
| All | <2.2e-16 |
| $bla_{KPC}$ vs $bla_{OXA-48}$-like | <2.2e-16 |
| $bla_{KPC}$ vs $bla_{VIM}$ | 1 |
| $bla_{OXA-48}$-like vs $bla_{VIM}$ | <2.2e-16 |
